# Applying Bipartite Network Analysis and Ordination Technique to Evaluate Long-Term Data from Veterinary–Sanitary Examination of Slaughtered Pigs

**DOI:** 10.3390/ani12040472

**Published:** 2022-02-14

**Authors:** Michał Majewski, Łukasz Dylewski, Sebastian Grabowski, Przemysław Racewicz, Piotr Tryjanowski

**Affiliations:** 1 Laboratory of Veterinary Public Health Protection, Poznań University of Life Sciences, Słoneczna 1, 62-002 Poznan, Poland; przemyslaw.racewicz@up.poznan.pl; 2 Department of Zoology, Poznań University of Life Sciences, Wojska Polskiego 71C, 60-625 Poznan, Poland; lukasz.dylewski@up.poznan.pl (Ł.D.); piotr.tryjanowski@gmail.com (P.T.); 3 Department of Epizootiology and Clinic of Infectious Diseases, Faculty of Veterinary Medicine, University of Life Sciences in Lublin, 20-400 Lublin, Poland; sebastian.grabowski@vp.pl

**Keywords:** veterinary inspection, meat inspection, heatmap, pig diseases, condemnation

## Abstract

**Simple Summary:**

Veterinary inspections in abattoirs are important in the surveillance of zoonotic diseases. This study is based on veterinary inspection reports providing data about the diseases and welfare issues of 231 million pigs slaughtered in 16 regions of Poland between 2009 and 2019. Over 80 million slaughtered pigs were found with pathological changes that could pose a risk to human health. The most frequently observed changes were abscesses, soiling, faecal or other contaminations, and congestions, together accounting for 77.6% of the total infections. Statistical approaches conducted with the use of long-term data can help identify the most problematic health and welfare issues in slaughterhouses. The results of this study showed that changes related to poor animal welfare (purulent foci, contamination, congestion) and parasitic diseases accounted for the majority of the identified cases of condemnation.

**Abstract:**

Animal and meat inspections in abattoirs are important in the surveillance of zoonotic diseases. Veterinary inspections in abattoirs can provide useful data for the management of health and welfare issues of humans and animals. Using the network analysis and ordination technique, in this study, we analyzed the data from 11 years of veterinary inspections in pig slaughterhouses from 16 regions in Poland. Based on the huge data set of 80,187,639 cases of diseases and welfare issues of pigs, the most frequent livestock diseases were identified to be abscesses, soiling, faecal or other contaminations, and congestions, which together accounted for 77.6% of the total condemnations. Spatial and temporal differences in swine diseases between the Polish regions were recognized using the above-mentioned statistical approaches. Moreover, with the use of a quite novel method, not used yet in preventive veterinary medicine, called a heatmap, the most problematic disease and welfare issues in each region in Poland were identified. The use of statistical approaches such as network analysis and ordination technique allow for identification of the health and welfare issues in slaughterhouses when dealing with long-term inspection data based on a very large number of cases, and then have to be adopted in current veterinary medicine.

## 1. Introduction

Foodborne diseases pose a significant threat to public health. It is estimated that every year about 10% of the world’s population becomes ill as a result of the consumption of unsafe foods [1]. Although Europe has the lowest incidence of foodborne diseases in the world, over 23 million people are affected due to the consumption of contaminated food each year [1]. Pork is a major source of animal protein accounting for over 36% of meat intake, and its consumption has increased by 13.8% in the last two decades [2]. Food safety can only be ensured by monitoring the welfare and health of animals during rearing, breeding, transport, and slaughter, as well as by maintaining a high level of hygiene while handling meat right from arrival to abattoirs until consumption and having a properly functioning surveillance system [3,4,5].

The inspection of animals and meat in abattoirs is important in the surveillance of zoonotic diseases. Meat has the high potential to be a significant source of infectious diseases. Rearing and breeding for meat production are associated with high epidemic risks [6]. Lack of hygiene in abattoirs and while cutting plants can lead to foodborne diseases [7]. Meat parasites are an important pathogen to be detected to protect public health. In addition, their detection is linked with significant economic losses caused by condemnations [8]. Pigs are a potential reservoir for many pathogenic organisms that can be transferred between animals and from animals to humans [9]. From animals, zoonotic agents can be transmitted to humans through direct contact or their tissues in the abattoir, or if meat containing the pathogens is consumed [10].

In recent years, researchers have been focusing more on issues related to animal welfare, but mass livestock farming does not allow to ensure optimal living conditions for animals. Injuries and lesions noted in animals during inspection can result in partial, or in severe cases, total carcass condemnation. Symptoms observed by Veterinary Food Inspectors during inspection can be valuable indicators of animal diseases as well as animal welfare [11]. Detection of pathological changes in abattoirs is particularly important because although official measures ensure the welfare of animals on the farms, they do not eliminate lesions in animals [12]. Therefore, antemortem and postmortem inspections carried out at abattoirs by Veterinary Food Inspectors are an important step toward ensuring food safety. Veterinary-sanitary inspection enables the elimination of diseased animals as well as the tissues obtained from these animals before they enter the food market. This method has also been used in studies on the veterinary-sanitary examination of slaughtered animals, with an aim of assessing the frequency of condemnations, their causes, and proportions [13].

Descriptive statistics (i.e., percentage of identified diseases) is the basis of classical approaches. It does not allow us to fully understand some dependencies arising because of the lack of a comprehensive approach. Conclusions drawn about pig health and welfare based on only quantitative or percentage data of individual diseases can be very broad and may only indicate changes in relative percentage [14,15]. In turn, large amounts of data on the number of cases of animal disease facilitate the analysis of the dynamics of change over time or the identification of a problem in a specific region. Therefore, veterinarians are searching for practical tools to improve the reaction to potential increases in numbers of diseases, which is a classical part of preventive veterinary medicine [3,12]. Among some others, detailed statistical analyses may help to find potential sources of infection, both on a spatial, as well as temporal scale. One possibility is the adoption of solutions known in other scientific disciplines. For example, statistical approaches such as network analysis and ordination technique, which are widely used in ecological studies, can also be applied for long-term data from the veterinary-sanitary examination. Network analysis is a useful technique for investigating interactions (i.e., plant-pollinators, plant–plant pest diseases) at the species level as well as at the community level [16]. This analysis is often used to determine the degree of specialization between partners based on indexes such as connectance, the number of links, and the specialization index (H2′). As veterinary inspection data provide enormous information about multi-disease cases from each region, the quasi-bipartite network analysis may be used to identify the (1) frequency of links between disease cases and regions and (2) differences in specialization index between years.

This study aimed to analyze the data from 11 years of veterinary inspection in pig slaughterhouses in all 16 Polish regions (voivodeships) representing a truly national study, using the network analysis and ordination technique.

## 2. Materials and Methods

### 2.1. Materials

**Figure 1:**
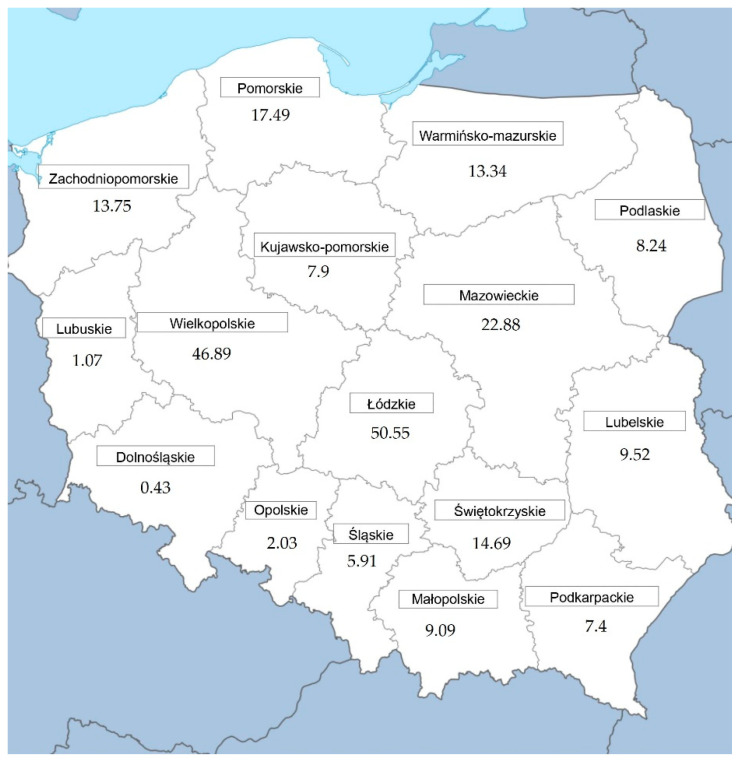
Geographical location of 16 voivodeships with the number of official pig examinations carried out in millions of heads in the period from 2009 to 2019.

The study used long-term veterinary inspection reports containing information about the diseases and welfare issues of 231 million pigs slaughtered in 16 regions of Poland between 2009 and 2019 (Figure 1). The data were collected by well-trained veterinary inspection authorities who conduct antemortem and postmortem inspections of slaughter animals, resulting in a huge database of livestock diseases. The authorities provide reports on the results of the official examination of animals and meat (RRW-6) every year. The data in the reports refer to lesions found in all species of slaughter animals. The official veterinarians in charge of supervision send an annual summary of antemortem and postmortem inspection results for each abattoir to the District Veterinary Officer (from 305 District Veterinary Inspectorates in Poland). The District Veterinary Officer aggregates the data for all abattoirs in the district and sends it to the Province Veterinary Officer (16 Province Veterinary Inspectorates in Poland). The General Veterinary Inspectorate receives data from all 16 provinces as an annual report (RRW-6). The report (RRW-6) is divided into two chapters. Chapter 1 of this report provides information about the number of antemortem and postmortem inspections conducted in the province. Chapter 2, in the case of postmortem inspection of pigs, provides data about infectious diseases detected in carcasses (e.g., tuberculosis, rubella, abscess, and septicemia), parasitic diseases (e.g., echinococcosis, trichinosis), and other noncontagious diseases and lesions.

### 2.2. Statistical Analysis

The effect of region and years, as well as the interaction between these two variables on the number of postslaughtered pig cases, was evaluated using linear regression. A heatmap was created based on the Pearson residuals to indicate the association of pig diseases with each region.

The composition of pig disease cases between regions was compared using nonmetric multidimensional scaling with the metaMDS function based on Bray–Curtis distance and three dimensions. The significance of fitted vectors was determined using permutation tests with 999 random data permutations. The permutational multivariate analysis of variance (PERMANOVA) was performed to check the differences between centroids and dispersion of groups representing each region, effect of years, and the interaction between them, using the adonis function in the vegan package [16]. Then, redundancy analysis (RDA) was carried out to verify the factors affecting pig disease cases. As explanatory variables, regions were included as a category variable and years as a continuous variable. A Monte Carlo test was performed in this analysis with 499 permutations to determine the significance of the canonical axes. Then, bipartite network analysis was performed, with the weighted matrix including the frequency of interaction of each disease case on each region.

The indices namely connectance, generality, vulnerability, nestedness, and degree of distribution were used in the qualitative analysis [17], while the indices namely weighted connectance, links per disease species with regions (links per species), weighted nested overlap, and decreasing fill (NODF), specialization asymmetry, and degree of complementary specialization (H2′ index) were used in the quantitative analysis. In addition, the specialization H2′ index was determined based on the weighted matrix including the frequency of interaction of each disease case on each region separately for each year. H2′ is a network-level index that is used for comparing different interaction webs. Its value ranges between 0 (no specialization) and 1 (perfect specialization). Finally, a simple linear regression model was constructed to verify the effect of years on the specialization index [3].

## 3. Results

During 2009–2019, a total of 332,700 (0.14%) pig carcasses were fully condemned due to the detection of lesions or other disqualifying changes in the meat. A total of 80,187,639 cases of diseases were reported during 11 years of veterinary inspections in 16 regions in Poland. The most frequently identified livestock diseases were abscesses, soiling, faecal or other contaminations, and congestions (accounting together for 77.6%), other diseases (e.g., adenomatosis, mycoplasmosis, pleuropneumonia) (accounting for 13.7%), and other parasite diseases (accounting for 7.3%) (Table A1).

The number of cases of disease was significantly different between regions (F = 16.59, *p <* 0.001) and between years (F = 4.34, *p =* 0.039). However, the changes in the number of postslaughter pig cases within years were dependent on the region (region × years interaction: F = 16.73, *p <* 0.001).

**Figure A1:**
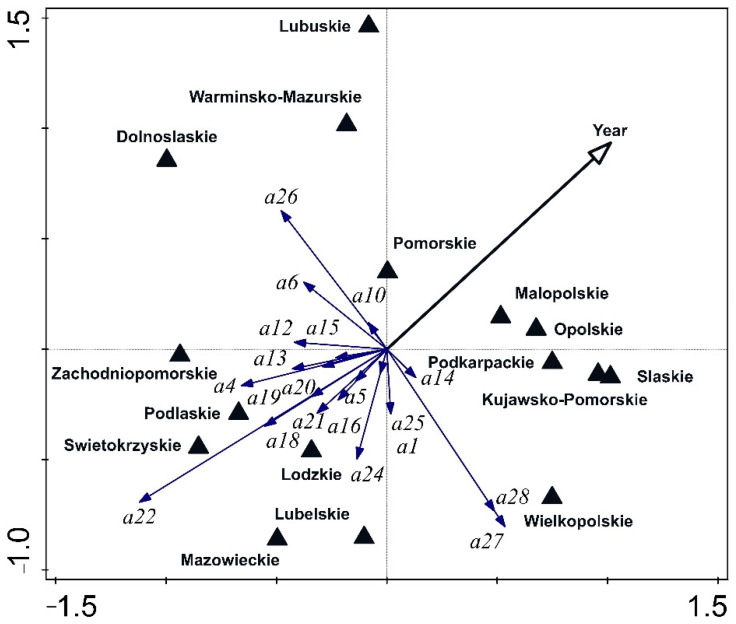
Ordination diagram of redundancy analysis of health and welfare issues in relation to 16 regions of Poland in 2009–2019. The abbreviation of health and welfare issues: a1—Tuberculosis; a2—Classical and African Swine fever; a3—Swine Pasteurellosis; a4—Swine Erysipelas; a5—Other Infectious Diseases; a6—Generalised Septicaemia and Pyaemia; a7—Salmonellosis; a8—Tetanus; a9—Actinomycosis; a10—Tumours; a11—Leukaemia; a12—Emaciated animals; a13—Jaundice; a14—Ascites; a15—Putrefaction; a16—Organoleptic Anomalies; a17—Contains Chemical Residues; a18—Insufficient Bleeding; a19—Dead Before Slaughter or Slaughter in Agony; a20—Trichinlellosis; a21—Cysticercosis; a22—Echinococcosis; a23—Fasciolosis; a24—Sarcosporidiosis; a25—Hypoplasia; a26—Purulent Foci, Contamination, Congestion; a27—Other Parasitic Diseases, a28—Other.

The PERMANOVA results showed significant differences in the types of pig diseases between regions (F = 10.66, *p =* 0.001, R^2^ = 43.7%). The years (F = 8.25, *p =* 0.002) and interaction between years and region (F = 3.57, *p =* 0.001) also had significant effects, but the differences accounted only for a lower percentage of variance (R^2^ = 2.3% and R^2^ = 14.7%, respectively). The results of RDA showed significant differences in the composition of disease cases (Monte Carlo test of the significance of the first and second axis: pseudo-F = 3.1, *p =* 0.002 and pseudo-F = 13.9, *p =* 0.002, respectively). The first and second canonical axes of RDA explained 43.14% of the variation in the composition of disease cases, of which 58.4% was related to explanatory variables. Forward selection of explanatory variables showed that regions and years had a significant effect on the composition of disease cases (Figure A1, Table A2).

**Figure 2:**
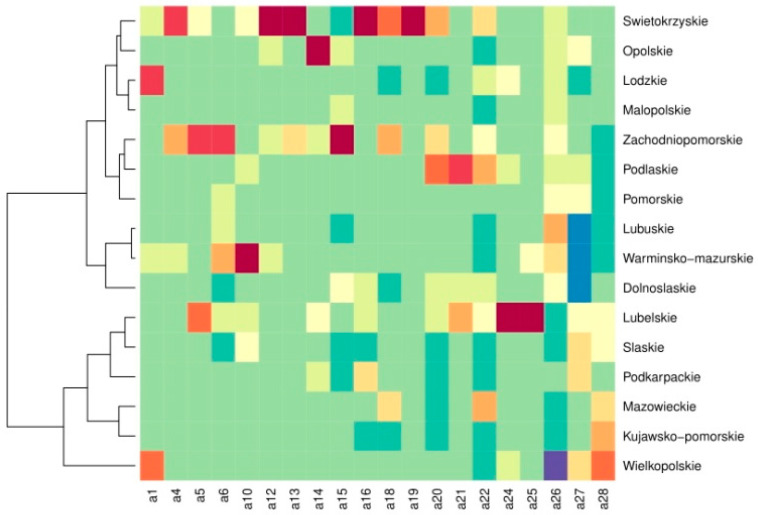
Heatmap based on the Pearson residuals of diseases and welfare issues in each region. Diseases that higher association with the region have red color in the heatmap, while those with lower association with the region are colored blue. The abbreviation of health and welfare issues: a1—Tuberculosis; a4—Swine Erysipelas; a5—Other Infectious Diseases; a6—Generalised Septicaemia and Pyaemia; a10—Tumours; a12—Emaciated Animals; a13—Jaundice; a14—Ascites; a15—Putrefaction; a16—Organoleptic Anomalies; a18—Insufficient Bleeding; a19—Dead Before Slaughter or Slaughter in Agony; a20—Trichinlellosis; a21—Cysticercosis; a22—Echinococcosis; a24—Sarcosporidiosis; a25—Hypoplasia; a26—Purulent Foci, Contamination, Congestion; a27—Other Parasitic Diseases, a28—Other.

The heatmap based on the Pearson residuals indicated the hotspots (i.e., higher associated) and cold spots (i.e., lower associated) of diseases as well as welfare issues in each region. For instance, the following were more frequent in the Swietokrzyskie region: emaciated animals, jaundice, organoleptic anomalies, and dead before slaughter or slaughter in agony. In the Lubuskie, Warminsko-Mazurskie, and Dolnoslaskie regions, other parasitic diseases were not observed (Figure 2).

**Figure 3:**
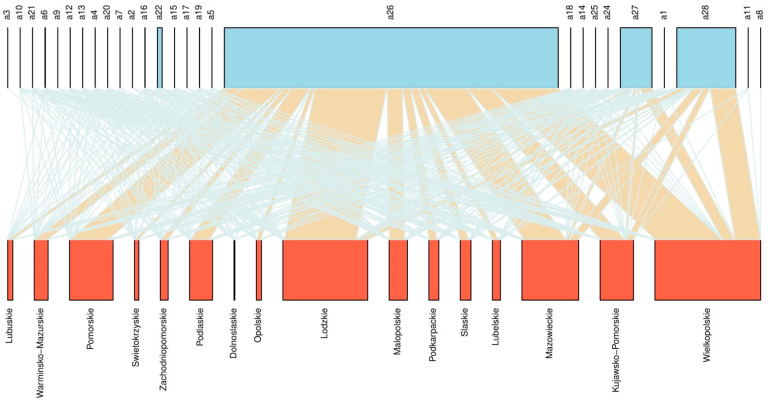
Bipartite network graph representing the links between total frequency of diagnosed diseases and welfare issues in 16 regions from 2009 to 2019 in Poland. The abbreviation of health and welfare issues: a1—Tuberculosis; a2—Classical and African Swine fever; a3—Swine Pasteurellosis; a4—Swine Erysipelas; a5—Other Infectious Diseases; a6—Generalised Septicaemia and Pyaemia; a7—Salmonellosis; a8—Tetanus; a9—Actinomycosis; a10—Tumours; a11—Leukaemia; a12—Emaciated animals; a13—Jaundice; a14—Ascites; a15—Putrefaction; a16—Organoleptic Anomalies; a17—Contains Chemical Residues; a18—Insufficient bleeding; a19—Dead Before Slaughter or Slaughter in Agony; a20—Trichinlellosis; a21—Cysticercosis; a22—Echinococcosis; a24—Sarcosporidiosis; a25—Hypoplasia; a26—Purulent Foci, Contamination, Congestion; a27—Other Parasitic Diseases, a28—Other.

**Figure A2:**
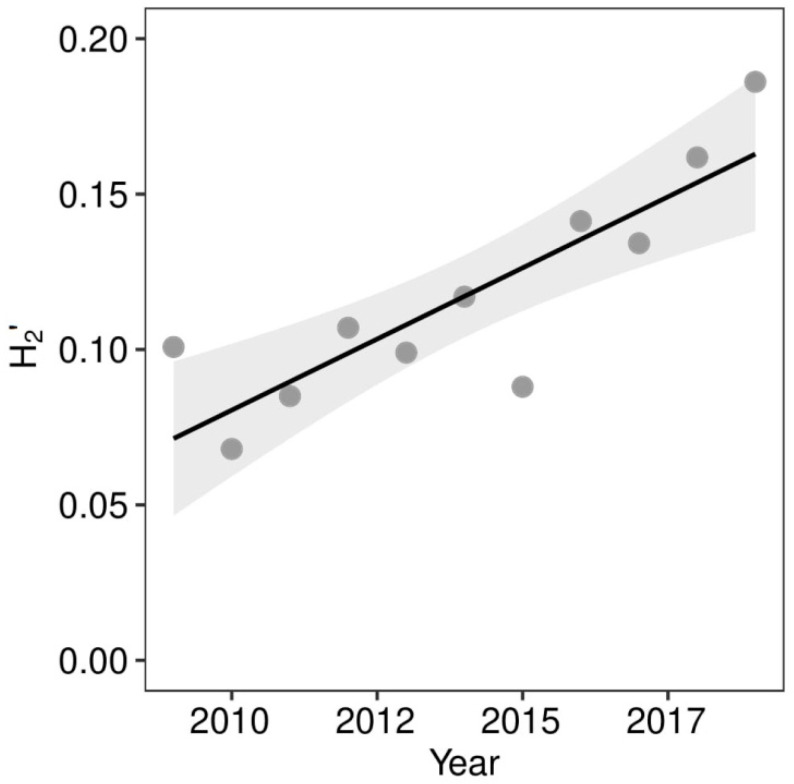
Relationship between years and specialization index.

The network analysis showed that the connectome was 0.69 and this metric was skewed toward generalization (>0.5). The same effect was also noted for other metrics, namely generality, vulnerability, or linkage density of weighted links (Table A3). Based on weighted links, the bipartite representations of region–pig disease case network are shown in Figure 3, with regions in the lower level and diseases in the upper level. The box size was proportional to the total number of disease cases recorded, and the breadth of the link to the frequency of association. The graph presents each pair of region and disease cases. It was found that echinococcosis, purulent foci, contamination, congestion, and other parasitic diseases were the most common in all regions. The degree of specialization based on the total number of disease cases in the whole year determined by the frequency-based index H2′ (0.069) was low. However, the analysis on separate years revealed that the specialization index increased significantly with the years (F_1,9_ = 24.37, R^2^ = 0.70, *p <* 0.001; Figure A2), which may indicate regional specialization for a given disease.

## 4. Discussion

According to the analyzed veterinary inspection reports in Poland, a total of 231,241,837 pig carcasses were inspected between 2009 and 2019. In 2021, there were 517 authorized slaughterhouses for pigs [18]. There is an increase in the number of pigs slaughtered in abattoirs in Poland and a related increase in the number of antemortem and postmortem inspections over the years. In 1994, more than 14 million pigs were slaughtered and 0.64% of them were found fully condemned [19]. In the analysis carried out by Szkucik et al. [20], it has been shown that between 2001 and 2011 the number of pigs slaughtered increased from 17.6 million units at the beginning of the 21st century to around 20 million by the end of the first decade. In the 11-year period covering the first decade, a total of 222 million pigs were tested, which proves that the number of slaughtered pigs in Poland has not changed in the second decade. Out of the postmortem inspected pig carcasses in 2001–2011, 97.2 million (43.76%) had pathological changes, and 342,777 pigs (0.15%) were fully condemned. There is an improvement in pig health in 2009–2019 compared to the first decade of the 21st century (43.76% in 2001–2011 vs. 34.67% in 2009–2019), but at the same time, there was no significant change in the number of carcasses considered fully condemned (0.15% vs. 0.14%) [20].

The Lodzkie and Wielkopolskie regions accounted for 21.86% and 20.28% of the total slaughtered pigs, respectively. It was found that the number of postslaughter pig cases differed between years depending on the region. Of the total slaughtered pigs, 34.7% were diagnosed with health or welfare issues. Veterinary inspections are necessary to ensure the health and welfare of pigs, which has an indirect impact on the quality of pork. Postmortem veterinary examinations of pigs conducted in slaughterhouses help in the detection of diseases in animals as well as poor animal welfare. The results of the study revealed that 77.6% of the slaughtered pigs had the most problematic issues (purulent foci, contamination, congestion), while 13.7% had the second frequently diagnosed health and welfare issues (e.g., adenomatosis, mycoplasmosis, pleuropneumonia).

The data revealed that 0.14% of pig carcasses were totally condemned in 2009–2019 in Poland, while in the United Kingdom only 0.003% were found to be unfit for consumption [21]. The season of the year can significantly influence the frequency of deaths before slaughter. In January, when the temperatures during transportation and in pens were lower, there were a higher number of antemortem rejections in Poland [22]. In an examination of slaughtered pigs in Portugal, 392 (0.24%) carcasses were found to be condemned due to osteomyelitis (38.5%), granulomatous lymphadenitis (22.7%), and pleurisy/pneumonia (21.2%) [22], while according to the analyzed data the most common causes of condemnations were sepsis and abscesses (34%), and abscesses, soiling, faecal or other contaminations, and congestions (24%). However, Guardone et al. [14] observed that in Tuscany (Italy), the total number of condemnations was greater in the summer and autumn, so it must be assumed that the number of condemnations varies based on the climatic zone and that critical weather conditions significantly affect the occurrence of lesions. Of the pigs slaughtered in 2010–2019 in Tuscany, 37.36% were condemned due to erysipelas and 26.07% due to generalized jaundice, while in the present study, there were only single cases of these conditions in all voivodeships (Table A1).

In Brazil, which is one of the world’s largest pork producers, abscesses were shown to be the most common lesions, accounting for 23.68–28.92% of the total condemnations in the years 2007–2009. The high number of condemnations was caused by technological factors. The most common management failures were death during transport (24.2–28.6%), emergencies (2.01–14.65%), and fractures/hematomas (4.6–26.71%), which accounted for 43.45%, 56.71%, and 55.66% in the following years of the study [23]. These findings suggest that poor welfare can result in a significant number of condemnations, implying that precise legal regulations and monitoring are critical. The European Union (EU) has been placing a high value on the protection of farm animals for decades. Due to the significant diversity of animal numbers and breeding methods in the member states, breeding issues are regulated by the directive, which allows individual countries to adjust internal regulations to achieve their goals [24]. During transport, many animals can be harmed or killed, and hence Regulation 1/2005 has been in force since 2005 to improve animal welfare at this stage [25]. Data on the deaths of animals transported to slaughterhouses provide evidence for the effectiveness of legal solutions. Of the pigs slaughtered (1.25 million) between 2010 and 2019 in Tuscany (Italy), 0.09% (1153) died during transport. As mentioned above, the season of the year can significantly influence the incidence of deaths. In summer, there were 35.17% of deaths, while in winter it was 16.53% [14]. Other studies also support this thesis, demonstrating that a higher number of deaths occur during transport in summer, while lesions occur more frequently in autumn [13]. The risk of death also increases during transportation if the animals are not starved before loading or if they are injured [26,27].

The present study showed that 7% of all condemnations were due to cachexia. Martínez et al. [28] reported that condemnation occurred more often in growth-retarded (usually intensively medicated) pigs. Among apparently healthy pigs, 0.5% were condemned completely and 0.2% partially. The most noted changes were bronchopneumonia (59.1%), pleuritis (17.2%), and pleuropneumonia (3.4%), which are respiratory system disorders. However, the causes of condemnation were vertebral osteomyelitis and abscesses. It was observed that 8.5% of carcasses of growth-retarded pigs were totally rejected, and 31% presented more than one lesion. Condemned carcasses were detected with abscesses (55.8%), cachexia (28.9%), catarrhal bronchopneumonia (16.2%), and vertebral osteomyelitis (9.6%). Other lesions occurred less frequently.

In Finland, over 95% of pig production is carried out by the system “Sikava”, which records data concerning antimicrobial treatment, biosecurity, welfare, and meat inspection scoring. It has been proved that adhering to biosecurity principles and ensuring a high level of animal welfare can improve meat quality [29]. Tail biting is an indicator of poor animal welfare. In herds where animals exhibit tail biting, the number of abscesses and arthritis cases are significantly higher [30]. Lesions related to poor welfare were less frequently observed in Finnish farms under the Sikava system. Abscesses were observed in 4.1% of herds and arthritis in 3.2% of herds in Finland. The most observed postmortem lesions were inflammatory lesions in the respiratory system, pleurisy (14.7%), and lung lesions (2%) [28].

The results of the present study showed spatial and temporal differences in the incidence of pathoanatomic findings in slaughterhouses. However, the effect of years accounted only for a low percentage of variance in each analysis. The RDA results indicated that the disease community significantly differed between the regions. Moreover, regions with similar cases of diseases were clustered. A heatmap created based on the Pearson residuals indicated the most serious pig condemnation cases in each region. This novel approach also allowed determining the condemnations that accounted for the highest and lowest percentage of cases in a given region. Diseases that have a higher association with the region have a red color in the heatmap, while those with a lower association with the region are coloured blue. Applying the bipartite network analyses in veterinary inspection can easily visualize the strength of the relationship between diseases and regions. Thanks to this, it is possible to identify the most serious diseases in pigs in a given region as well as to quickly identify coexisting diseases, which allows for quick prevention by veterinary inspection. Network analysis indicated that the specialization index significantly differed between years, which affected the regionalization for a given disease. The food choices of consumers are increasingly influenced by the welfare of food-producing animals. In turn, the approaches of the meat industry are determined by customer expectations. In a study in the EU, 94% of respondents reported that it is important to protect the welfare of all farmed animals, while 59% declared that they would pay a slightly higher price for food produced from animals with a higher level of welfare. However, if the price of food was increased by more than 20%, only 3% were willing to pay for it [31]. This suggests that although consumers’ awareness about animal welfare is increasing, animal welfare does not yet play a significant role in food choices. In addition, there are significant differences in how different countries perceive the well-being of animals. Consumers in Germany value factors such as animal-friendly rearing systems, shorter transport time, and use of anesthetics during castration of piglets, whereas consumers in Poland value factors associated with food safety such as the absence of pathogens (*Salmonella*), use of feed free from genetically modified organisms, and traceability in the food chain.

## 5. Conclusions

The present study on slaughterhouse pigs based on long-term data can contribute to a better understanding of pig health and welfare as well as the identification of pathoanatomic conditions in each region in Poland. The analysis of long-term data revealed that poor animal welfare (i.e., purulent foci, contamination, congestion), as well as parasitic diseases, accounts for the highest percentage of all identified cases. This raises concerns about the confinement of animals in farms or the improper transport of animals to slaughterhouses. Moreover, the heatmap approach helped in the identification of the most serious pig diseases and welfare issues in each region. The ASF epidemic in Poland and the whole of Eastern Europe resulted in an increase in the frequency and intensity of veterinary controls in farms keeping pigs. This is an excellent opportunity to put emphasis on animal welfare in addition to biosecurity issues. Understanding the patterns of swine disease and welfare issues is crucial for the swine industry’s decision-making. Our proposed statistic tools for analyzing big veterinary inspection data could help to cost-effective approaches to monitor, prevent, control, and eradicate infectious diseases that occur in slaughtered pigs.

## Data Availability

The data presented in this study are available on request from the corresponding author.

## Appendix A

**Table A1:** Percentage of health and welfare issues in slaughterhouses in 16 regions of Poland in 2009–2019.

Disease	Dolnoslaskie	Kujawsko-Pomorskie	Lubelskie	Lubuskie	Lodzkie	Malopolskie	Mazowieckie	Opolskie	Podkarpackie	Podlaskie	Pomorskie	Slaskie	Swietokrzyskie	Warminsko-Mazurskie	Wielkopolskie	Zachodniopomorskie
Tuberculosis	0.0000	0.0005	0.0000	0.0000	0.0058	0.0000	0.0000	0.0000	0.0001	0.0000	0.0001	0.0000	0.0006	0.0014	0.0057	0.0000
Classical swine fever and African swine fever	0.0000	0.0000	0.0000	0.0000	0.0000	0.0001	0.0000	0.0000	0.0000	0.0000	0.0000	0.0000	0.0000	0.0000	0.0000	0.0000
Swine pasteurellosis	0.0000	0.0000	0.0000	0.0000	0.0000	0.0000	0.0000	0.0000	0.0000	0.0000	0.0000	0.0000	0.0000	0.0000	0.0000	0.0000
Swine erysipelas	0.0026	0.0076	0.0111	0.0085	0.0173	0.0072	0.0165	0.0003	0.0150	0.0189	0.0264	0.0021	0.2295	0.0508	0.0195	0.2086
Other infectious diseases	0.0000	0.0001	0.1416	0.0000	0.0000	0.0169	0.0000	0.0001	0.0000	0.0001	0.0000	0.0000	0.0440	0.0001	0.0100	0.2248
Generalized septicemia and pyemia	0.0154	0.0172	0.1896	0.2173	0.0177	0.0110	0.0331	0.1441	0.0438	0.0531	0.1845	0.0023	0.0995	0.5393	0.0667	0.8952
Salmonellosis	0.0000	0.0000	0.0000	0.0001	0.0001	0.0000	0.0000	0.0000	0.0000	0.0000	0.0000	0.0007	0.0000	0.0000	0.0000	0.0000
Tetanus	0.0000	0.0000	0.0000	0.0000	0.0000	0.0000	0.0000	0.0000	0.0000	0.0000	0.0000	0.0000	0.0000	0.0000	0.0000	0.0000
Actinomycosis	0.0000	0.0000	0.0000	0.0000	0.0001	0.0001	0.0000	0.0000	0.0000	0.0000	0.0000	0.0000	0.0000	0.0000	0.0000	0.0000
Tumours	0.0000	0.0000	0.0001	0.0000	0.0000	0.0000	0.0000	0.0000	0.0001	0.0001	0.0001	0.0002	0.0001	0.0007	0.0000	0.0000
Leukemia	0.0000	0.0000	0.0001	0.0000	0.0000	0.0000	0.0000	0.0000	0.0000	0.0000	0.0000	0.0000	0.0000	0.0000	0.0000	0.0000
Emaciated animals	0.0071	0.0036	0.0177	0.0471	0.0109	0.0053	0.0136	0.0494	0.0073	0.0256	0.0178	0.0079	0.2234	0.0569	0.0142	0.0667
Jaundice	0.0026	0.0023	0.0287	0.0144	0.0036	0.0012	0.0078	0.0130	0.0119	0.0151	0.0290	0.0023	0.1800	0.0185	0.0117	0.0877
Ascites	0.0006	0.0004	0.0047	0.0001	0.0009	0.0012	0.0003	0.0154	0.0022	0.0009	0.0003	0.0004	0.0018	0.0009	0.0011	0.0016
Putrefaction	0.0006	0.0000	0.0001	0.0000	0.0001	0.0004	0.0001	0.0003	0.0000	0.0001	0.0000	0.0000	0.0000	0.0000	0.0001	0.0018
Organoleptic anomalies	0.0863	0.0073	0.1661	0.0167	0.0593	0.0277	0.0574	0.0550	0.1713	0.0257	0.0273	0.0056	0.3860	0.0478	0.0303	0.0486
Presence of chemical residues	0.0000	0.0000	0.0000	0.0000	0.0000	0.0000	0.0000	0.0000	0.0001	0.0000	0.0000	0.0000	0.0000	0.0000	0.0000	0.0000
Insufficient bleeding	0.0051	0.0059	0.0267	0.0110	0.0056	0.0107	0.0675	0.0119	0.0186	0.0211	0.0125	0.0107	0.0995	0.0180	0.0119	0.0910
Dead before slaughter or slaughter in agony	0.0200	0.0138	0.0181	0.0217	0.0207	0.0427	0.0171	0.0277	0.0018	0.0310	0.0042	0.0055	0.4749	0.0223	0.0232	0.0163
Trichinellosis	0.0006	0.0001	0.0004	0.0001	0.0001	0.0001	0.0001	0.0002	0.0000	0.0014	0.0005	0.0001	0.0006	0.0002	0.0003	0.0012
Cysticercosis	0.0071	0.0006	0.0155	0.0000	0.0004	0.0000	0.0000	0.0001	0.0000	0.0192	0.0008	0.0006	0.0001	0.0004	0.0001	0.0001
Echinococcosis	1.6100	0.0232	1.7295	0.0308	1.5044	0.1345	3.1709	0.0594	0.0559	2.8131	0.2893	0.0197	2.6883	0.1397	0.3652	1.7411
Fasciolosis	0.0000	0.0000	0.0000	0.0000	0.0000	0.0000	0.0000	0.0000	0.0000	0.0000	0.0000	0.0000	0.0000	0.0000	0.0000	0.0000
Sarcosporidiosis	0.0000	0.0001	0.0178	0.0000	0.0034	0.0000	0.0001	0.0000	0.0002	0.0024	0.0001	0.0001	0.0000	0.0013	0.0029	0.0001
Hypoplasia	0.0000	0.0002	0.1493	0.0000	0.0003	0.0041	0.0023	0.0000	0.0000	0.0000	0.0005	0.0019	0.0082	0.0486	0.0087	0.0008
Purulent foci, contamination, congestion	86.4563	74.6081	73.5204	96.7781	84.9158	83.9157	74.4262	84.8928	80.5288	82.3864	88.7560	77.7611	85.9834	94.4428	63.1623	86.3848
Other parasitic diseases	1.6100	4.8916	10.0008	0.4849	3.9438	5.5463	4.6349	7.5888	10.7306	11.5842	8.4924	9.9891	4.3984	1.2363	11.8230	5.2216
Other	10.1756	20.4174	13.9616	2.3693	9.4896	10.2747	17.5520	7.1414	8.4124	3.0014	2.1582	12.1899	5.1816	3.3741	24.4429	5.0080

**Table A2:** Results of forward selection analysis of explanatory variables in RDA, explaining the patterns of disease case composition in the 11 years of veterinary inspection. The analysis was performed using Monte Carlo tests with 499 permutations. Variables are ordered according to their stepwise inclusion into the model. Statistically significant effects are shown in bold (*p* < 0.05).

Name of Variable	Pseudo-*F*	*p*-Value
region.Dolnoslaskie	13.4	0.002
region.Lubuskie	14.2	0.002
region.Swietokrzyskie	13.2	0.002
region.Zachodniopomorskie	15.4	0.002
region.Warminsko-Mazurskie	15.3	0.002
Year	10.3	0.002
region.Mazowieckie	10.3	0.002
region.Podlaskie	12.6	0.002
region.Pomorskie	11.2	0.002
region.Lodzkie	10.7	0.002
region.Lubelskie	13.9	0.002
region.Wielkopolskie	4.6	0.002
region.Kujawsko-Pomorskie	3.2	0.014
region.Slaskie	2.3	0.054
region.Malopolskie	1.6	0.162
region.Podkarpackie	0.9	0.496

**Table A3:** Values of the indices determined from the weighted links of regions–disease network from 2009 to 2019 based on the veterinary inspection in Poland.

Connectance	0.689
Links per species	6.93
Weighted NODF	63.24
Specialization asymmetry	0.277
Shannon diversity	2.93
H2′	0.069
Mean number of shared partners	7.63
Generality	9.17
Interaction strength asymmetry	0.027
Interaction evenness	0.483
**Specialization index**	
H2′	0.069
H2min	2.27
H2max	2.97
H2obs	2.93
